# Obesity and loss of disease-free years owing to major non-communicable diseases: a multicohort study

**DOI:** 10.1016/S2468-2667(18)30139-7

**Published:** 2018-09-01

**Authors:** Solja T Nyberg, G David Batty, Jaana Pentti, Marianna Virtanen, Lars Alfredsson, Eleonor I Fransson, Marcel Goldberg, Katriina Heikkilä, Markus Jokela, Anders Knutsson, Markku Koskenvuo, Tea Lallukka, Constanze Leineweber, Joni V Lindbohm, Ida E H Madsen, Linda L Magnusson Hanson, Maria Nordin, Tuula Oksanen, Olli Pietiläinen, Ossi Rahkonen, Reiner Rugulies, Martin J Shipley, Sari Stenholm, Sakari Suominen, Töres Theorell, Jussi Vahtera, Peter J M Westerholm, Hugo Westerlund, Marie Zins, Mark Hamer, Archana Singh-Manoux, Joshua A Bell, Jane E Ferrie, Mika Kivimäki

**Affiliations:** a Clinicum, Department of Public Health, Faculty of Medicine, University of Helsinki, Helsinki, Finland; b Department of Psychology and Logopedics, Faculty of Medicine, University of Helsinki, Helsinki, Finland; c Department of Epidemiology and Public Health, University College London, London, UK; d Department of Public Health, University of Turku and Turku University Hospital, Turku, Finland; e Finnish Institute of Occupational Health, Helsinki, Finland; f Institute of Public Health and Caring Sciences, Uppsala University, Uppsala, Sweden; g Department of Medical Sciences, Uppsala University, Uppsala, Sweden; h Stress Research Institute, University of Stockholm, Stockholm, Sweden; i Centre for Occupational and Environmental Medicine, Stockholm County Council, Sweden; j Institute of Environmental Medicine, Karolinska Institutet, Stockholm, Sweden; k School of Health and Welfare, Jönköping University, Jönköping, Sweden; l Paris Descartes University, Paris, France; m Inserm UMS 011, Population-Based Epidemiological Cohorts Unit, Villejuif, France; n Department of Health Services Research and Policy, London School of Hygiene & Tropical Medicine, London, UK; o Clinical Effectiveness Unit, The Royal College of Surgeons, London, UK; p Department of Health Sciences, Mid Sweden University, Sundsvall, Sweden; q National Research Centre for the Working Environment, Copenhagen, Denmark; r Department of Psychology, Umeå University, Umeå, Sweden; s Department of Public Health and Department of Psychology, University of Copenhagen, Copenhagen, Denmark; t Faculty of Social Sciences (Health Sciences), University of Tampere, Tampere, Finland; u University of Skövde, School of Health and Education, Skövde, Sweden; v National Centre for Sport and Exercise Medicine, Loughborough University, Loughborough, UK; w Inserm U1018, Centre for Research in Epidemiology and Population Health, Villejuif, France; x MRC Integrative Epidemiology Unit, University of Bristol, Bristol, UK; y Bristol Medical School: Population Health Sciences, University of Bristol, Bristol, UK

## Abstract

**Background:**

Obesity increases the risk of several chronic diseases, but the extent to which the obesity-related loss of disease-free years varies by lifestyle category and across socioeconomic groups is unclear. We estimated the number of years free from major non-communicable diseases in adults who are overweight and obese, compared with those who are normal weight.

**Methods:**

We pooled individual-level data on body-mass index (BMI) and non-communicable diseases from men and women with no initial evidence of these diseases in European cohort studies from the Individual-Participant-Data Meta-Analysis in Working Populations consortium. BMI was assessed at baseline (1991–2008) and non-communicable diseases (incident type 2 diabetes, coronary heart disease, stroke, cancer, asthma, and chronic obstructive pulmonary disease) were ascertained via linkage to records from national health registries, repeated medical examinations, or self-report. Disease-free years from age 40 years to 75 years associated with underweight (BMI <18·5 kg/m^2^), overweight (≥25 kg/m^2^ to <30 kg/m^2^), and obesity (class I [mild] ≥30 kg/m^2^ to <35 kg/m^2^; class II–III [severe] ≥35 kg/m^2^) compared with normal weight (≥18·5 kg/m^2^ to <25 kg/m^2^) were estimated.

**Findings:**

Of 137 503 participants from ten studies, we excluded 6973 owing to missing data and 10 349 with prevalent disease at baseline, resulting in an analytic sample of 120 181 participants. Of 47 127 men, 211 (0·4%) were underweight, 21 468 (45·6%) normal weight, 20 738 (44·0%) overweight, 3982 (8·4%) class I obese, and 728 (1·5%) class II–III obese. The corresponding numbers among the 73 054 women were 1493 (2·0%), 44 760 (61·3%), 19 553 (26·8%), 5670 (7·8%), and 1578 (2·2%), respectively. During 1 328 873 person-years at risk (mean follow-up 11·5 years [range 6·3–18·6]), 8159 men and 8100 women developed at least one non-communicable disease. Between 40 years and 75 years, the estimated number of disease-free years was 29·3 (95% CI 28·8–29·8) in normal-weight men and 29·4 (28·7–30·0) in normal-weight women. Compared with normal weight, the loss of disease-free years in men was 1·8 (95% CI −1·3 to 4·9) for underweight, 1·1 (0·7 to 1·5) for overweight, 3·9 (2·9 to 4·9) for class I obese, and 8·5 (7·1 to 9·8) for class II–III obese. The corresponding estimates for women were 0·0 (−1·4 to 1·4) for underweight, 1·1 (0·6 to 1·5) for overweight, 2·7 (1·5 to 3·9) for class I obese, and 7·3 (6·1 to 8·6) for class II–III obese. The loss of disease-free years associated with class II–III obesity varied between 7·1 and 10·0 years in subgroups of participants of different socioeconomic level, physical activity level, and smoking habit.

**Interpretation:**

Mild obesity was associated with the loss of one in ten, and severe obesity the loss of one in four potential disease-free years during middle and later adulthood. This increasing loss of disease-free years as obesity becomes more severe occurred in both sexes, among smokers and non-smokers, the physically active and inactive, and across the socioeconomic hierarchy.

**Funding:**

NordForsk, UK Medical Research Council, US National Institute on Aging, Academy of Finland, Helsinki Institute of Life Science, and Cancer Research UK.

## Introduction

Obesity is a growing public health problem.^1^, ^2^ In 2016, more than 1·9 billion adults worldwide were overweight and 650 million were obese, vastly outnumbering those who were normal weight.^3^ In addition to reducing quality of life^4^ and life expectancy,^5^ obesity is associated with an elevated risk of several major non-communicable diseases, including type 2 diabetes, coronary heart disease, stroke, asthma, and several cancers.^6^, ^7^, ^8^ Early studies suggested that metabolically healthy obesity, especially when combined with a high level of fitness, is associated with only a minimal increase in disease risk,^9^ but more recent longitudinal studies have shown that when examining individual change over time, healthy obese adults show a strong tendency to progress to an unhealthy obese state.^10^, ^11^

**Research in context**

**Evidence before this study**

We searched for studies examining the association between obesity and disease-free years in PubMed and Embase, without language or date restrictions up to March 1, 2018, using the terms “obesity”, “body mass index”, “healthy years”, “healthy life-years”, “disease-free years”, “disease-free life expectancy”, “healthy life expectancy”, “life expectancy”. We found evidence linking obesity with increased risk of single chronic conditions, but few studies quantified the mean number of disease-free years that obese individuals lose compared with those of healthy weight.

**Added value of this study**

To our knowledge, this is the largest study so far to examine the association between obesity and loss of disease-free years due to major non-communicable diseases and how this association varies by lifestyle category and socioeconomics. We found that mild obesity was associated with a loss of one in ten and severe obesity a loss of one in four potential disease-free years between 40 years and 75 years. In both sexes, overweight participants lost 1 disease-free year, the mildly obese 3–4 years, and the severely obese 7–8 years compared with normal-weight participants. Severe obesity was associated with a loss of 7–10 disease-free years in active and inactive individuals; current, past, and never smokers; and those of high and low socioeconomic position.

**Implications of all the available evidence**

Quantifying the number of obesity-related disease-free years lost is important for the development of prevention strategies for major non-communicable chronic diseases in health policies and clinical guidelines. Our findings suggest that the substantial loss of disease-free years associated with obesity is evident across the social hierarchy and irrespective of lifestyle factors, such as physical activity and smoking.

Several studies have documented associations between obesity and risk of major non-communicable diseases, based on risk relative to normal weight using body-mass index (BMI) categories. By contrast, few studies have quantified such associations using absolute metrics such as disease-free years.^5^, ^12^, ^13^, ^14^, ^15^ Furthermore, those that have used absolute metrics have tended to use narrow and varying definitions of disease-free years, such as free of cardiovascular disease^12^, ^14^, ^15^ or free of cardiometabolic disease,^5^ or have been based on mainly self-reported data.^13^ Given this heterogeneity in the definition of disease-free years and assessment methodology, the loss of years free of major non-communicable diseases attributable to overweight and obesity remains ill defined. Physical exertion might mitigate the adverse effects of obesity,^16^, ^17^, ^18^ but to our knowledge no study has estimated the extent to which physical activity helps to decrease the obesity-related loss of disease-free years. Similarly for smoking habits and socioeconomic position, evidence is sparse.

In the present study, we aimed to calculate the loss of disease-free years in adults who are overweight and obese, compared with those who are normal weight.

## Methods

### Study population

We used data from independent European studies from the Individual-Participant-Data Meta-Analysis in Working Populations (IPD-Work) consortium.^19^ All participating cohort studies have been approved by local ethics committees, and details of study designs and participants are described in the appendix (pp 2–4). Written informed consent was obtained from all participants.

### Procedures

We calculated BMI as weight in kg divided by height in m^2^. Participants with missing values for height or weight or BMI values less than 15 kg/m^2^ or more than 50 kg/m^2^ were excluded, as per previous analyses. We classified BMI into five categories according to WHO recommendations: underweight (<18·5 kg/m^2^), normal weight (≥18·5 kg/m^2^ to <25 kg/m^2^), overweight (≥25 kg/m^2^ to <30 kg/m^2^), class I (mild) obesity (≥30 kg/m^2^ to <35 kg/m^2^), and class II–III (severe) obesity (≥35 kg/m^2^).

Socioeconomic group was based on occupational title obtained from employers' or other registers or questionnaires completed by participants or self-reported highest educational qualification. For each study, socioeconomic group was categorised as high (e.g. professionals or executives), intermediate (e.g. skilled non-manual workers), or low (e.g. manual workers; appendix p 4).

Individuals were classified as never, former, or current smokers on the basis of information extracted from participant questionnaires in all studies. Physical activity at baseline was self-reported and participants were categorised as physically active if they engaged in at least moderate levels of activity, and inactive otherwise. A detailed description of the baseline assessment is available in the appendix (pp 3–4).

Linked records of non-communicable diseases covered both baseline and follow-up. The outcome of interest was the first record of incident type 2 diabetes, coronary heart disease, stroke, cancer, asthma, or chronic obstructive pulmonary disease (COPD), because these are the commonest major non-communicable diseases in developed countries.^20^ These non-communicable diseases are also targets prioritised for global disease prevention by WHO.

Type 2 diabetes was identified via hospital discharge registers and mortality registers as the appearance of E11 (International Classification of Diseases, revision 10 [ICD-10]) or 250 (ICD-9) in any of the diagnosis codes; 2-h oral glucose tolerance tests administered every 5 years (fasting glucose ≥7·0 mmol/L or 2-h post-load glucose ≥11·1 mmol/L); the first time the participant appeared in the nationwide drug reimbursement register as eligible for medication for this condition; or self-report from annual questionnaires.

Coronary heart disease was identified from hospital discharge and mortality registers, annual self-report questionnaires, or clinical screening using WHO Multinational Monitoring of Trends and Determinants in Cardiovascular Disease (MONICA) Project criteria.^21^ We included all non-fatal myocardial infarctions recorded as I21–I22 (ICD-10) or 410 (ICD-9), and coronary deaths I20–I25 (ICD-10) and 410–414 (ICD-9) in the diagnosis codes. Incident stroke was ascertained via hospital and mortality records: I60, I61, I63, and I64 (ICD-10); and 430, 431, 433, 434, and 436 (ICD-9).

Cancers, C00–C97 (ICD-10 any cancer), were identified via national cancer or mortality registers, the employer's medical register, or by confirming any self-reported cancer diagnosis with the participant's physician. Severe asthma (J45 or J46 in ICD-10 or 493 in ICD-9) in any diagnostic code and COPD exacerbations (J41, J42, J43, and J44 in ICD-10, or 491, 492, and 496 in ICD-9) were ascertained from hospital discharge and death registers in all studies except for one study, in which non-fatal cancer and asthma events were based on self-report from annual questionnaires and non-fatal COPD was not available. Detailed descriptions of the outcome measurements are available in the appendix (pp 6–7).

Participants with missing data on outcomes and those with a record of any of these diseases already at baseline were excluded from the analyses. We also excluded participants with a record of type 1 diabetes at baseline: E10 (ICD-10) or 250 (ICD-9 and ICD-8).^22^

### Statistical analysis

We did all analyses separately for men and women. Disease-free years were defined as the number of life-years between ages 40 years and 75 years free from diagnosis of any of the non-communicable diseases examined. To estimate the association between BMI category and disease-free years, hazard ratios (HRs) with 95% CIs for the first disease were calculated using flexible parametric survival models on the cumulative hazards scale.^23^ Restricted cubic splines were fitted within these models to model the baseline hazard for each BMI category using age as the timescale. Disease-free years lost associated with overweight and obesity compared with normal weight were calculated as the difference between the areas under the disease-free survival curves from ages 40 years to 75 years. Area under the curve was computed via numerical integration with a spline-based method. Disease-free years were estimated conditional on survival to age 40 years without any of the six major non-communicable diseases investigated. We chose 40 years because this is typically the age at which health checks, monitoring of specific cancers, and assessment of risk of chronic conditions, such as cardiovascular disease, commences.^24^, ^25^, ^26^ CIs for disease-free years were estimated via bootstrapping using 1000 independent replications. We used two-stage meta-analysis to combine the results. The results were first calculated separately within each study (first stage), then the study-specific results were pooled using random effects meta-analysis (second stage). Heterogeneity between cohorts was assessed with the *I*^2^ statistic.

To examine effect modification, we stratified the analyses by categories of physical activity, smoking, and socioeconomic position. To achieve sufficient power for these subgroup analyses, we pooled the data and used study identifier as a covariate in the model. A sensitivity analysis was done for the main model, comparing the results of the pooled analyses to those obtained from the two-stage models to justify data pooling.

Data were analysed using Stata/MP 13.1, packages stpm2 and metan.^27^, ^28^

### Role of the funding source

The funders of the study had no role in study design, data collection, data analysis, data interpretation, or writing this report. STN and MKi had full access to data from FPS, HeSSup, Gazel, Whitehall II, WOLF N, and WOLF S cohort studies; MV had full access to data from the SLOSH study; TL had full access to data from HHS; and IEHM had full access to data from the DWECS and IPAW studies. STN and MKi had final responsibility for the decision to submit for publication.

## Results

**Figure 1:**
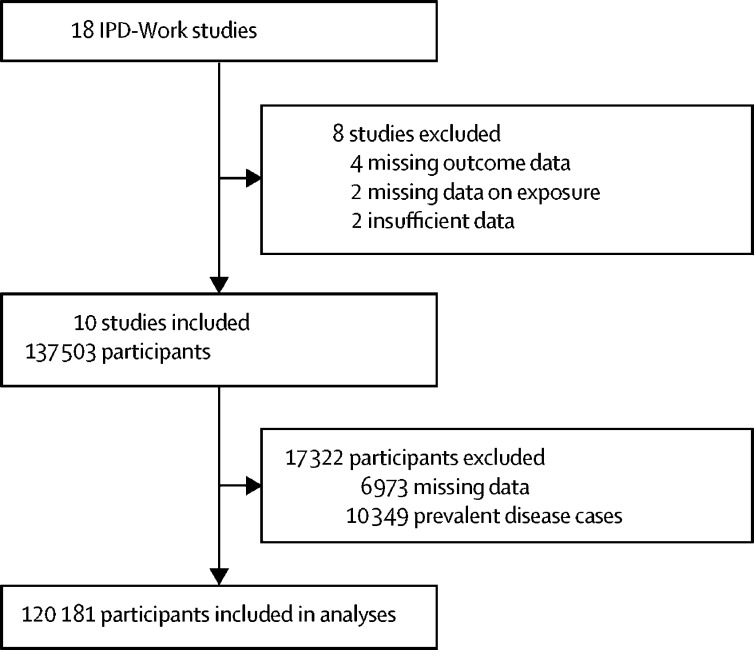
Study profile IPD-Work=Individual-Participant-Data Meta-Analysis in Working Populations.

Of the 18 IPD-Work cohort studies, eight were excluded owing to missing data on exposure or outcome, or insufficient data (to ensure participant confidentiality) for the analyses (figure 1). Study baseline varied between 1991 and 2008 depending on the cohort. Of the 137 503 participants from ten IPD-Work cohort studies, we excluded 6973 owing to missing data on age, sex, BMI, or any of the non-communicable diseases investigated and 10 349 with a recorded history of these diseases at baseline, resulting in an analytic sample of 120 181 participants (figure 1). 73 054 (60·8%) participants were women and 47 127 (39·2%) were men. Mean age at baseline was 44·6 years (SD 9·7) for men and 43·4 years (9·9) for women. For men, mean BMI was 25·7 kg/m^2^ (SD 3·4), with 211 (0·4%) underweight, 21 468 (45·6%) normal weight, 20 738 (44·0%) overweight, 3982 (8·4%) class I obese, and 728 (1·5%) class II–III obese. For women, mean BMI was 24·5 kg/m^2^ (SD 4·0), with 1493 (2·0%) underweight, 44 760 (61·3%) normal weight, 19 553 (26·8%) overweight, 5670 (7·8%) class I obese, and 1578 (2·2%) class II–III obese. Other characteristics of the analytic sample are presented in the appendix (p 5).

Mean follow-up was 11·5 years (range between studies 6·3–18·6), with 1 328 873 person-years at risk. 8159 men had at least one incident disease during 543 522 person-years at risk; the corresponding figure was 8100 for women during 785 350 person-years at risk (appendix p 7).

**Figure 2:**
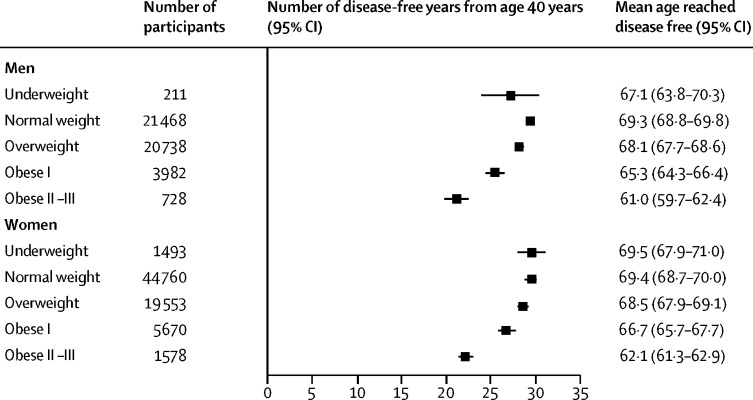
Number of disease-free years from age 40 years by BMI category BMI=body-mass index.

Normal-weight men had a mean of 29·3 disease-free years (95% CI 28·8–29·8) between 40 years and 75 years (figure 2). The estimate for underweight participants was 27·1 disease-free years (95% CI 23·8–30·3). In women, the corresponding estimates were 29·4 disease-free years (95% CI 28·7–30·0) for normal weight and 29·5 disease-free years (27·9–31·0) for underweight.

Compared with normal weight, the loss of disease-free years for underweight was 1·8 (95% CI −1·3 to 4·9) in men and 0·0 (−1·4 to 1·4) for women. Above normal weight, the number of disease-free years diminished with increasing BMI category. Compared with normal-weight men, overweight men lost 1·1 disease-free years (95% CI 0·7–1·5), class I obese men lost 3·9 disease-free years (2·9–4·9), and class II–III obese men lost 8·5 disease-free years (7·1–9·8). The corresponding reductions for women were 1·1 disease-free years (95% CI 0·6–1·5) for overweight, 2·7 disease-free years (1·5–3·9) for obese class I, and 7·3 disease-free years (6·1–8·6) for obese class II–III. The appendix (pp 8–9) provides study-specific estimates of the numbers of disease-free years by BMI category.

**Figure 3:**
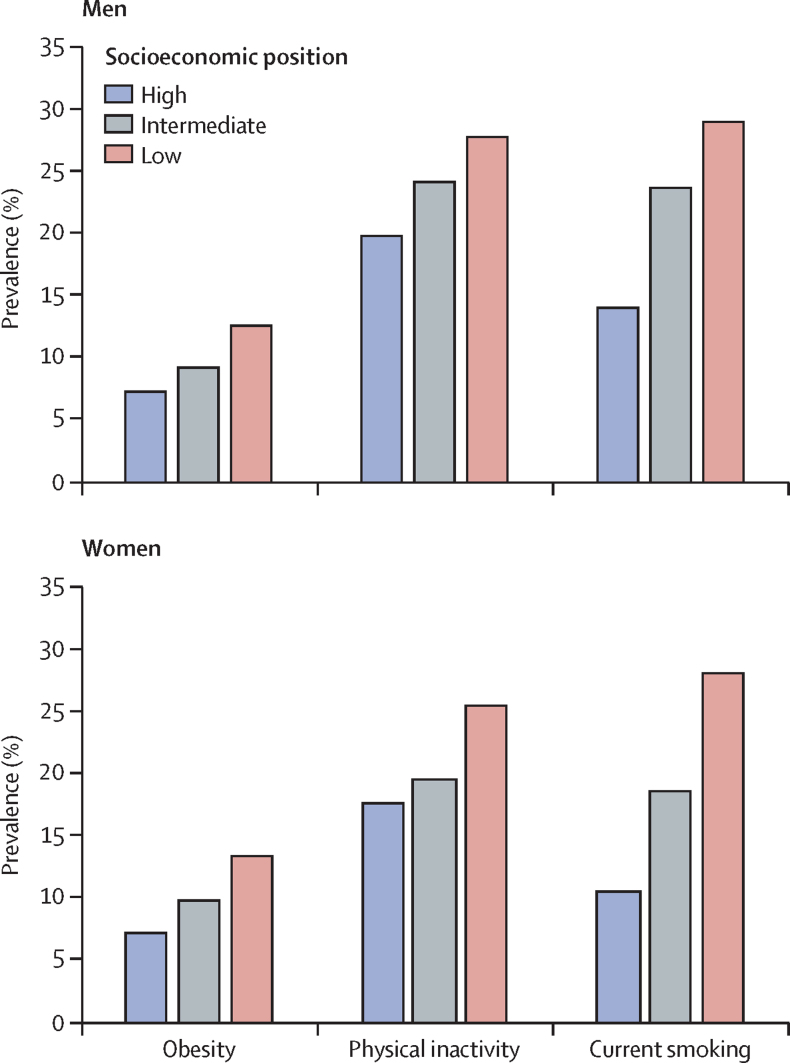
Prevalence of obesity, physical inactivity, and smoking by socioeconomic position

**Figure 4:**
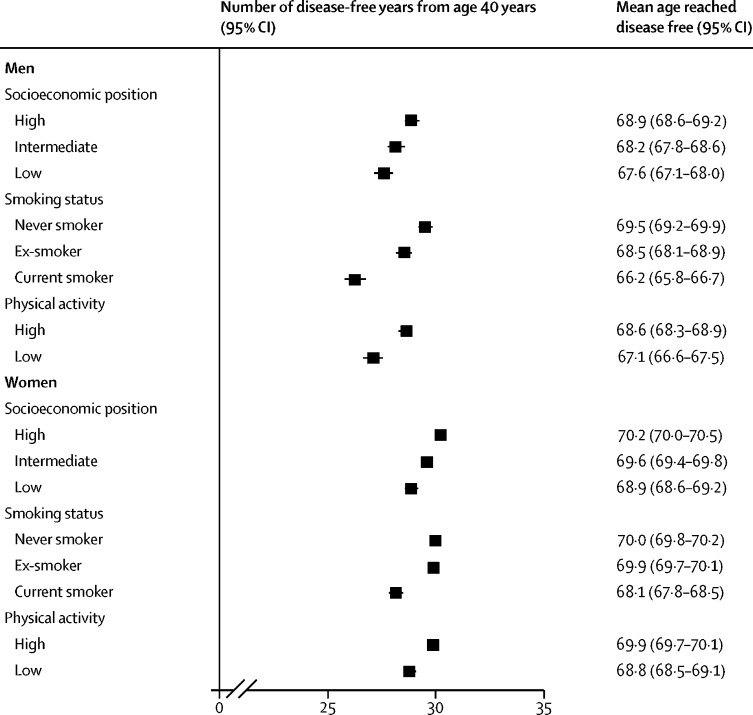
Number of disease-free years from age 40 years by socioeconomic position, smoking status, and physical activity level

Supplementary and subgroup analyses are based on a pooled dataset and are shown in the appendix (pp 10–11, 14–15). Four studies (DWECS, IPAW, SLOSH, and HHS) were not included in the pooled dataset because no individual-level data were provided by the investigators. Obesity, physical inactivity, and smoking were socially patterned, with higher prevalences in lower socioeconomic groups (figure 3). Low socioeconomic group, smoking, and physical inactivity were associated with fewer disease-free years (figure 4).

**Figure 5:**
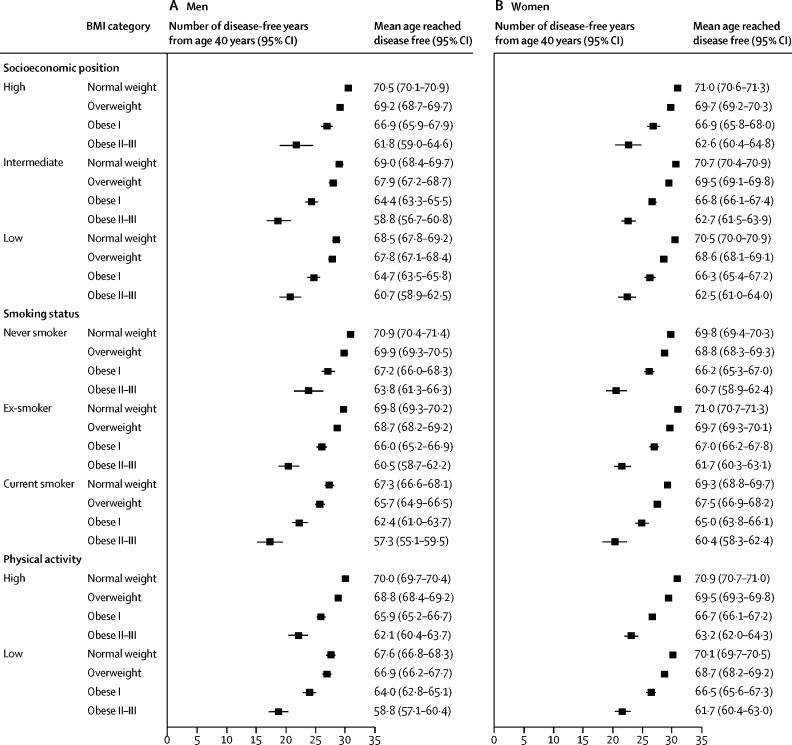
Subgroup analyses of number of disease-free years from age 40 years by BMI category BMI=body-mass index.

To examine whether the association between BMI and disease-free years was independent of the effects of lifestyle risk factors and their socioeconomic patterning, stratified analyses were done. The association between BMI category and the loss of disease-free years was replicated at each level of the socioeconomic hierarchy (figure 5). Compared with normal-weight men, those with class II–III obesity lost 8·7 disease-free years (95% CI 6·0–11·4) among those in a high socioeconomic position and 7·8 disease-free years (6·0–9·5) among those in a low socioeconomic position. Corresponding losses in class II–III obese women were 8·3 disease-free years (95% CI 6·1–10·6) for high and 8·0 disease-free years (6·5–9·5) for low socioeconomic position participants.

Class II–III obese men who were current smokers had 10·0 fewer disease-free years (95% CI 7·9–12·2) than normal-weight men (figure 5). The corresponding losses among class II–III obese men were 9·3 disease-free years (95% CI 7·6–11·0) for past smokers and 7·1 disease-free years (4·8–9·3) for never smokers. Among class II–III obese women compared with normal weight, current smokers lost 8·9 disease-free years (95% CI 6·8–11·0), past smokers lost 9·3 disease-free years (7·9–10·7), and never smokers lost 9·2 disease-free years (7·5–10·8).

Compared with normal-weight men, those with class II–III obesity who were physically active lost 8·0 disease-free years (95% CI 6·4–9·6) and inactive men lost 8·8 disease-free years (7·2–10·4; figure 5). Corresponding losses in class II–III obese women were 7·7 disease-free years (95% CI 6·5–8·9) for physically active and 8·4 disease-free years (7·2–9·7) for inactive participants.

## Discussion

Our results from over 120 000 Europeans show that, compared with normal weight, both mild and severe obesity are associated with a significant reduction in disease-free years between 40 years and 75 years. This finding was observed in both sexes, smokers and non-smokers, active and inactive people, and across the social hierarchy, suggesting that this association is ubiquitous and not limited to any specific group of people. Compared with their normal-weight counterparts, severely obese men and women lost about 7–8 disease-free years, whereas those who were mildly obese lost 3–4 disease-free years, and those who were overweight lost 1·1 disease-free years. Our findings suggest that mildly obese individuals lose one in ten and the severely obese one in four potential disease-free life-years during middle and later adulthood.

We observed socioeconomic gradients in obesity, physical activity, and smoking, in both men and women. Similarly, we observed strong associations between disease-free years and socioeconomic position, physical activity, and smoking. Despite these relationships, loss of disease-free years associated with obesity varied little between socioeconomic groups and those with more and less favourable lifestyle factors. The loss of disease-free years associated with class I obesity was about 4 years in both physically active and inactive participants and varied between 2 years and 5 years among current smokers, past smokers, and non-smokers and those from high and low socioeconomic backgrounds. The loss of disease-free years associated with class II–III obesity was greater, between 7 years and 10 years, among participants with different physical activity levels, smoking habits, and socioeconomic positions. These findings suggest that the link between BMI and the number of disease-free years is robust, adding to previous evidence on the adverse associations of obesity on disease-free lifespan.^5^, ^12^, ^13^, ^14^, ^15^

Our analyses benefit from the large sample size and the fact that in nine of the ten studies non-communicable diseases were defined using electronic health records from national health registers. At least one previous multicohort study^13^ has reported a link between obesity and the loss of expected disease-free years, although that study was smaller, defined the outcome based on self-rated data, did not report summary data from the four studies included, and covered a shorter age range (50–75 years). Several other studies have also concluded that obese adults have fewer disease-free years than normal-weight adults.^5^, ^12^, ^15^ One exception is a study of 19 420 adults, which found that overweight was associated with a greater number of disease-free years compared with normal weight and that obese participants had a similar number of disease-free years compared with normal-weight participants.^14^ Such findings are in contrast to those of large collaborative observational and instrumental variable studies, which reported a steadily increased risk of cardiovascular disease incidence^29^ and all-cause and cause-specific mortality^30^ above normal weight for height, suggesting that the apparently protective associations of overweight for healthy lifespan are driven by confounding or bias.

The current findings on obesity are biologically plausible. For example, high levels of free fatty acids, inflammatory cytokines, lipid intermediates, and insulin resistance with excess total and intra-abdominal adipose tissue are among the pathophysiological mechanisms^31^ underlying increased type 2 diabetes risk among obese people.^32^ Obesity-related excess adipose tissue surrounding and compressing the kidney, along with overactivity of the sympathetic nervous system contribute to hypertension, which is an important pathophysiological mechanism underlying heart diseases, stroke, and chronic kidney diseases.^32^ High fasting insulin concentrations^33^, ^34^ together with increased lipid concentrations and lipid signalling can fuel cancer pathogenesis, and a low-grade inflammatory response might accelerate cancer progression.^35^ Obesity produces changes in immune function, which affect host defence and might also have implications for immune diseases such as asthma.^36^

The difference in the number of disease-free years between underweight and normal weight was small. Other studies have shown that underweight is strongly associated with poor health and lower survival,^37^ but in those reports the participants were not initially free of major non-communicable conditions, as was the case in our study. Thus, the proportion of participants underweight due to weight loss caused by underlying chronic disease is likely to be greater in those investigations than in our study. Furthermore, many of the studies included in the present consortium were occupational cohort studies, which include healthier people than the general population, further increasing the proportion of healthy underweight participants.

This study has several limitations. First, there was heterogeneity in some study-specific estimates, potentially attributable to differences in assessment methods, settings, and variable definitions. Second, we limited loss of disease-free years to the commonest major chronic diseases: type 2 diabetes, coronary heart disease, stroke, cancer, asthma, and COPD. These are prioritised targets for prevention of premature mortality, although some cancers and COPD are not causally related to adiposity. We excluded conditions considered as risk factors for major non-communicable diseases, such as hypertension and chronic kidney disease, and less fatal obesity-related disorders, such as musculoskeletal problems or obstructive sleep apnoea.^38^, ^39^, ^40^, ^41^ Thus, the observed loss of disease-free years is attributable to specific major conditions and reflects excess risk in a group of people with obesity rather than a loss of disease-free years caused by obesity. Third, although ascertainment of major non-communicable diseases using linkage to electronic health records is comprehensive in relation to cancer, stroke, and coronary heart disease (as defined by myocardial infarction and coronary death), records of type 2 diabetes, asthma, and COPD in hospital and death registries cover only severe cases as these chronic diseases are typically diagnosed and treated in primary care and do not require hospital admissions. As expected, the incidence of each chronic disease was higher in men than women. However, women were diagnosed on average at a younger age than men, a finding that might partially relate to sex differences in health-care seeking behaviours.^42^

Further limitations are crude measurements of physical activity and socioeconomic position and, in some cohort studies, the use of self-reported rather than measured height and weight to calculate BMI. BMI is only an indirect measure of total body fat,^43^ and our findings were based on a single assessment of BMI, although repeat measurement from childhood to adulthood could have more comprehensively captured any additional effects of childhood obesity on the associations between BMI and number of disease-free years in adulthood. These limitations are more likely to lead to an underestimation than overestimation of the association between obesity and disease-free years. Finally, we cannot rule out the possibility of some residual confounding and reverse causality, despite subgroup analyses and the exclusion of participants with extant disease at baseline.

Despite these drawbacks, our results provide consistent evidence of an association between obesity and loss of disease-free years that exists in men and women, irrespective of position in the social hierarchy and lifestyle factors, such as smoking or physical activity. These findings lend support to obesity prevention as an important strategy for the reduction of morbidity.
