# Supplementary material for: Obesity and loss of disease-free years owing to major non-communicable diseases: a multicohort study
Source: Lancet Public Health. 2018 Sep 1;3(10):e490–7. doi: 10.1016/S2468-2667(18)30139-7 (PMC6178874; doi:10.1016/S2468-2667(18)30139-7)

THE LANCET
Public Health

Supplementary appendix

This appendix formed part of the original submission and has been peer reviewed. We post it as supplied by the authors.

Supplement to: Nyberg ST, Batty GD, Pentti J, et al. Obesity and loss of disease-free years owing to major non-communicable diseases: a multicohort study. *Lancet Public Health* 2018; published online Aug 31. http://dx.doi.org/10.1016/S2468-2667(18)30139-7.

Supplementary appendix

Supplement to: Nyberg ST, Batty GD, Pentti J, et al. Obesity and loss of disease-free years owing to major non-communicable diseases: a multicohort study. *Lancet Public Health*.

Contents

	Page
**Appendix 1: Description of the included studies**	2
Details of the study design and participants	2
Baseline assessments	3
Supplement Table 1. Baseline characteristics of the participants	5
**Appendix 2: Noncommunicable diseases and disease-free life-years**	6
Ascertainment of major noncommunicable diseases during follow-up	6
Supplemental Table 2. Incidence and mean age of diagnosis for individual noncommunicable diseases during follow-up in the pooled data	7
**Appendix 3: Study-specific analyses**	8
Supplemental Figure 1. Study-specific estimates for men	8
Supplemental Figure 2. Study-specific estimates for women	9
**Appendix 4: Sensitivity and supplementary analyses**	10
Supplement Table 3. Comparison of the mean number of disease-free years after age 40 from the two-stage analysis and the pooled analysis	11
Supplementary Figure 3. Country-specific estimates for the number of disease-free life-years after age 40 by BMI category for men	12
Supplementary Figure 4. Country-specific estimates for the number of disease-free life-years after age 40 by BMI category for women	13
Supplemental Table 4. Number of disease-free life-years after age 40 by BMI category before and after excluding non-melanoma skin cancer has been excluded from the outcome	14
Supplementary Table 5. Loss of disease-free years in underweight, overweight and obese participants compared to those with normal by smoking status, physical activity and socioeconomic position	15
Supplementary Figure 5. Loss of disease-free years in overweight and obese participants compared to those with normal in studies with self-reported height and weight and those with measured height and weight	16

Appendix 1: Description of the included studies

Details of the study design and participants

Danish Work Environment Cohort Study (DWECS), Denmark
DWECS is a split panel survey of working age Danish people. The cohort was established in 1990, when a simple random sample of men and women, aged 18-59, was drawn from the Danish population register. The participants have been followed up at five year intervals and data from the year 2000 was used for the IPD-Work. That year 11 437 individuals were invited to participate and 8 583 agreed to do so.[1] In Denmark, questionnaire- and register-based studies do not require ethics committee approval. DWECS was approved by and registered with the Danish Data protection agency (registration number: 2007-54-0059). Participants were provided information about the study with the baseline questionnaire and responding was taken to imply informed consent to take part.

Finnish Public Sector study (FPS), Finland
The Finnish Public Sector study is a prospective cohort study comprising the entire public sector personnel of 10 towns (municipalities) and 21 hospitals in the same geographical areas. Participants, who were recruited from employers' records in 2000-2002, were individuals who had been employed in the study organisations for at least six months prior to data collection.[2] 48 592 individuals (9 337 men and 39 255 women aged 17 to 65) responded to the questionnaire. Ethical approval was obtained from the ethics committee of the Finnish Institute of Occupational Health. According to the Finnish law, written consent is not required for survey and register-based research, as long as that participation is voluntary, and the participants have been informed about the aims of the study and the possible register linkages. Thus, responding to the questionnaire voluntarily (having had access to information on the study aims and possible register linkages) was taken to imply written consent.

Gazel, France
Gazel is a prospective cohort study of 20 625 employees (15 011 men and 5 614 women) of France's national gas and electricity company, Electricité de France-Gaz de France (EDF-GDF) [3,4] . Since the study baseline in 1989, when the participants were aged 35–50 years, they have been posted an annual follow-up questionnaire to collect data on health, lifestyle, individual, familial, social, and occupational factors. Gazel in 1997 was treated as a baseline year for our analyses. 11 448 individuals participated that year. The GAZEL study received approval from the national commission overseeing ethical data collection in France (Commission Nationale Informatique et Liberté). Participants in GAZEL did not fill in any formal informed consent. However, CNIL (the French legal authority for data privacy) considered that as participants themselves fill in and send written questionnaires, this is equivalent to a formal consent.

Health and Social Support (HeSSup), Finland
The Health and Social Support (HeSSup) study is a prospective cohort study of a stratified random sample of the Finnish population in the following four age groups: 20–24, 30–34, 40–44, and 50–54. The participants were identified from the Finnish population register and posted an invitation to participate, along with a baseline questionnaire, in 1998.[5] 25 898 individuals responded to the questionnaire in 1998. The Turku University Central Hospital Ethics Committee approved the study. All participants gave written informed consent to take part.

Helsinki Health Study (HHS), Finland
The Finnish Helsinki Health Study (HHS) is a prospective cohort study comprising all employees of the City of Helsinki, who turned 40, 45, 50, 55, or 60 years in 2000-2002.[6] We included in this study all participants who responded to the baseline survey (n=8960, response rate 67%, 80% women) and provided an informed written consent to combine their survey responses with retrospective and prospective register based follow-up data on different diseases and mortality (n=6605). Ethical approvals for this study were obtained from the ethics committees of the health authorities of the City of Helsinki, and the Department of Public Health, University of Helsinki.

Intervention Project on Absence and Well-being (IPAW), Denmark
IPAW is a 5-year psychosocial work environment intervention study including 22 intervention and 30 control work places in three organisations (a large pharmaceutical company, municipal technical services and municipal nursing homes) in Copenhagen, Denmark.[7] The baseline questionnaire was posted to all the employees at the selected work-sites between 1996 and 1997. Of the 2 721 employees who worked at the 52 IPAW sites, 2 068 men and women completed the baseline questionnaire. Interventions took place at 22 workplaces during 1996-98 at the organisational and interpersonal level. IPAW was approved by and registered with the Danish Data Protection Agency (registration number: 2000-54-0066). Participants were provided information about the study and with the baseline questionnaire and responding was taken to imply informed consent to take part.

Swedish Longitudinal Occupational Survey of Health (SLOSH), Sweden
Swedish Longitudinal Occupational Survey of Health (SLOSH) is an on-going prospective cohort study following up individuals who participated in the Swedish Work Environment Survey (SWES) in 2003 or 2005. SWES, conducted biennially by Statistics Sweden, is based on a sample of gainfully employed people aged 16-64 years drawn from the Labour Force Survey (LFS). These individuals were first sampled into LFS through stratification by county, sex, citizenship and inferred employment status.

Data from the 2006 and 2008 data collection waves of SLOSH were used in the IPD-Work analyses.[8] In both years, data were collected using postal self-completion questionnaires. In 2006, 5 985 individuals responded to the questionnaires. In 2008, a further 6 751 individuals responded to the questionnaires. SLOSH has been approved by the Regional Research Ethics Board in Stockholm. The participants received written information about the study and, in accordance with Swedish regulation and practice, responding to and returning the survey indicated informed consent.

Whitehall II, UK
The Whitehall II study is a prospective cohort study set up to investigate socioeconomic determinants of health. At study baseline in 1985-1988, 10 308 civil service employees (6 895 men and 3 413 women) aged 35-55 and working in 20 civil service departments in London were invited to participate in the study [9]. The Whitehall II study protocol was approved by the University College London Medical School committee on the ethics of human research. Written informed consent was obtained at each data collection wave.

WOLF (Work, Lipids, and Fibrinogen) Stockholm and WOLF Norrland studies, Sweden
The WOLF (Work, Lipids, and Fibrinogen) Stockholm study is a prospective cohort study of 5 698 people (3 239 men and 2 459 women) aged 19–70 and working in companies in Stockholm county [10]. WOLF Norrland is a prospective cohort of 4 718 participants aged 19-65 working in companies in Jämtland and Västernorrland counties [11]. At study baseline the participants underwent a clinical examination and completed a set of health questionnaires. For WOLF Stockholm, the baseline assessment was undertaken at 20 occupational health units between November 1992 and June 1995 and for WOLF Norrland at 13 occupational health service units in 1996-98. The Regional Research Ethics Board in Stockholm, and the ethics committee at Karolinska Institutet, Stockholm, Sweden approved the study. The participants received written and verbal information about the study and participation was voluntary. Answering the baseline questionnaire was taken to imply informed consent to participate.

Baseline assessments

We calculated BMI as weight in kilograms divided by height in meters squared. Height and weight were measured (Whitehall II, WOLF N and WOLF S) or self-reported (DWECS, FPS, Gazel, HeSSup, HHS, IPAW and SLOSH). Participants with missing values for height or weight or BMI values <15 or >50 kg/m2 were excluded, as per prior analyses.[12]

We classified BMI into five categories according to World Health Organization (WHO) recommendations.[13] Participants with a BMI < 18·5 kg/m^2 were categorized as underweight, those with a BMI between 18·5 and <25 kg/m^2 were denoted as normal weight, and those with a BMI between 25 and <30 kg/m^2 as overweight.

We included two subcategories of obesity: class I (BMI 30 to <35 kg/m^2) and class II and class III combined (BMI ≥35 kg/m^2).

Socioeconomic position was based on occupational title obtained from employers' or other registers (DWECS, FPS, Gazel, HHS and IPAW) or questionnaires completed by participants (HeSSup, SLOSH, Whitehall II, WOLF N and WOLF S).[14] In HeSSup, we used participants' self-reported highest educational qualification. For each study, socioeconomic position was categorised into low, intermediate, or high.

Individuals were classified as never, former or current smokers based on information extracted from participant questionnaires in all studies.[15] Physical activity at baseline was self-reported and differed between studies.[16] Some studies only had questions on sports activities and exercise, while for other studies information was also available for other types of leisure-time physical activities, such as walking and cycling. We constructed a measure of physical inactivity defined as no or very little, moderate or vigorous physical activity or exercise based on the best available information in each study. Examples of definitions of physical inactivity are "no weekly leisure-time physical activity," "no or very little exercise, only occasional walks," and "sport activities a few times per year or less." Individuals were categorized as physically active if they engaged in at least moderate levels of activity.[16]

References

1. Feveile H, Olsen O, Burr H, Bach E. Danish Work Environment Cohort Study 2005: From idea to sampling design. *Statistics in Transition* 2007; **8**(3): 441-58.
2. Kivimäki M, Lawlor DA, Smith GD, et al. Socioeconomic Position, Co-Occurrence of Behavior-Related Risk Factors, and Coronary Heart Disease: the Finnish Public Sector Study. *Am J Public Health* 2007; **97**(5): 874-9.
3. Goldberg M, Leclerc A, Bonenfant S, et al. Cohort profile: the GAZEL Cohort Study. *Int J Epidemiol* 2007; **36**(1): 32-9.
4. Zins M, Leclerc A, Goldberg M. The French GAZEL Cohort Study: 20 Years of Epidemiologi Research. *Advances in Life Course Research* 2009; **14**: 135-46.
5. Korkeila K, Suominen S, Ahvenainen J, et al. Non-response and related factors in a nation-wide health survey. *Eur J Epidemiol* 2001; **17**(11): 991-9.
6. Lahelma E, Aittomaki A, Laaksonen M, et al. Cohort profile: the Helsinki Health Study. *Int J Epidemiol* 2013; **42**(3): 722-30.
7. Nielsen M, Kristensen T, Smith-Hansen L. The Intervention Project on Absence and Well-being (IPAW): design and results from the baseline of a 5-year study. *Work and Stress* 2002; **16**: 191-206.
8. Magnusson Hanson LL, Leineweber C, Persson V, Hyde M, Theorell T, Westerlund H. Cohort Profile: The Swedish Longitudinal Occupational Survey of Health (SLOSH). *Int J Epidemiol* 2018.
9. Marmot MG, Smith GD, Stansfeld S, et al. Health inequalities among British civil servants: the Whitehall II study. *Lancet* 1991; **337**(8754): 1387-93.
10. Peter R, Alfredsson L, Hammar N, Siegrist J, Theorell T, P. W. High effort, low reward, and cardiovascular risk factors in employed Swedish men and women: baseline results from the WOLF Study. *J Epidemiol Community Health* 1998; **52**: 540-7
11. Alfredsson L, Hammar N, Fransson E, et al. Job strain and major risk factors for coronary heart disease among employed males and females in a Swedish study on work, lipids and fibrinogen. *Scand J Work Environ Health* 2002; **28**(4): 238-48.
12. Nyberg ST, Heikkilä K, Fransson EI, et al. Job strain in relation to body mass index: pooled analysis of 160 000 adults from 13 cohort studies. *J Intern Med* 2012; **272**(1): 65-73.
13. Obesity: preventing and managing the global epidemic. Report of a WHO consultation. World Health Organization technical report series 2000; **894**: i-xii, 1-253.
14. Kivimäki M, Virtanen M, Kawachi I, et al. Long working hours, socioeconomic status and the risk of incident type 2 diabetes: Meta-analysis of published and unpublished data from 222,120 individuals. *Lancet Diab Endocrinol* 2015;**3**:27-34.
15. Heikkilä K, Nyberg ST, Fransson EI, et al. Job strain and tobacco smoking: An individual-participant data meta-analysis of 166 130 adults in 15 European studies. *PloS ONE* 2012; **7**(7): e35463.
16. Fransson EI, Heikkila K, Nyberg ST, et al. Job strain as a risk factor for leisure-time physical inactivity: An individual-participant meta-analysis of up to 170,000 men and women: The IPD-Work Consortium. *Am J Epidemiol* 2012; **176**(12): 1078-89.

Supplemental table 1 Baseline characteristics of the participants from 10 prospective cohort studies

Study	Country	Baseline year	Sex	Number of participants	Mean (SD) age, years	Mean (SD) BMI, kg/m^2
DWECS	Denmark	2000	Men	3875	42·1 (13·7)	25.3 (3·5)
			Women	3997	41·9 (13·6)	23.7 (3·9)
FPS	Finland	2000	Men	8510	44·9 (9·4)	26·0 (3·5)
			Women	36137	44·4 (9·4)	24·7 (4·0)
Gazel	France	1997	Men	6947	51·0 (2·4)	26·0 (3·0)
			Women	2635	48·3 (3·6)	23·5 (3·7)
HeSSup	Finland	1998	Men	8962	37·3 (11·4)	25·2 (3·6)
			Women	12 900	35·9 (11·4)	23·9 (4·1)
HHS	Finland	2000, 2001, 2002	Men	1308	49·8 (6·6)	26·3 (3·8)
			Women	4857	49·1 (6·6)	25·3 (4·3)
IPAW	Denmark	1996-1997	Men	630	41·1 (10·0)	25·5 (3·3)
			Women	1279	40·9 (10·5)	23·5 (3·7)
SLOSH	Sweden	2006 and 2008	Men	4934	47·5 (11·4)	26·0 (3·4)
			Women	6055	46·7 (11·6)	24·7 (4·0)
Whitehall II	UK	1991-1993	Men	5139	49·1 (5·9)	25·1 (3·1)
			Women	2246	50·0 (6·1)	25·6 (4·7)
WOLF N	Sweden	1996-1998	Men	3727	43·6 (10·3)	26·3 (3·5)
			Women	700	44·2 (10·0)	25·4 (4·1)
WOLF S	Sweden	1992-1995	Men	3095	41·4 (11·1)	25·2 (3·3)
			Women	2248	40·9 (10·8)	23·8 (3·7)
**Total**		1991-2008	Men	47 127	44·6 (9·7)	25·7 (3·4)
			Women	73 054	43·4 (9·9)	24·5 (4·0)

Abbreviations. DWECS, Danish Work Environment Cohort Study; FPS, the Finnish Public Sector Study; Gazel, a cohort study of Électricité de France-Gaz de France employees; HeSSup, the Health and Social Support Cohort Study; HHS, Helsinki Health Study; IPAW, Intervention Project on Absence and Well-being; SLOSH, Swedish Longitudinal Occupational Survey of Health; Whitehall II, the Whitehall II Study; WOLF N, the Work, Lipids and Fibrinogen Study, Norrland; WOLF S, the Work, Lipids and Fibrinogen Study, Stockholm.

Appendix 2: Definition of disease-free life-years

Ascertainment of major noncommunicable diseases during follow-up

The outcome of interest in the present study was the first record of either incident type 2 diabetes, CHD, stroke, cancer, asthma or COPD. Participants with missing data on outcomes and participants with a record of any of these diseases at baseline were excluded from the analyses. We additionally excluded participants with a record of type 1 diabetes at baseline E10 (International Classification of Disease, revision 10 [ICD-10]) or 250 (ICD-9 and ICD-8).[1]

Incident type 2 diabetes was identified via hospital discharge registers and mortality registers as the appearance of E11 (ICD-10) or 250 (ICD-9) in any of the diagnosis codes. Additionally, in the Finnish datasets (FPS, HeSSup, and HHS), incident type 2 diabetes was identified the first time the participant appeared in the nationwide drug reimbursement register as eligible for medication for this condition.2 In the Whitehall II study, type 2 diabetes was ascertained via 2-hour oral glucose tolerance test administered every 5 years[3] using World Health Organization criteria and complemented by self-reports of diabetes diagnosis and medication.[4] In the Gazel study, non-fatal cases were based on self-report from annual questionnaires.[1]

Incident CHD and stroke during follow-up was identified from hospital discharge and mortality registers in all studies, except for Gazel, in which hospital register data were not available and non-fatal events were collected from annual self-report questionnaires. Incident non-fatal myocardial infarction or fatal coronary heart disease was defined by using the World Health Organization MONICA (Multinational Monitoring of Trends and Determinants in Cardiovascular Disease) Project criteria.[5] We included all non-fatal myocardial infarctions recorded as I21–I22 (ICD-10) or 410 (ICD-9), and coronary deaths I20–I25 (ICD-10) and 410–414 (ICD-9) in any of the diagnosis codes. Incident stroke was ascertained via hospital and mortality records; I60, I61, I63, I64 (ICD-10), 430, 431, 433, 434, 436 (ICD-9).[6,7]

Incident cancers, C00-C97 (ICD-10 any cancer) were identified via national cancer or mortality registers, except for Gazel, in which they were ascertained from the employer's medical register or by confirming any self-reported cancer diagnosis with the participant's physician.[8]

Severe asthma and COPD exacerbations were ascertained from hospital discharge and death registers in all studies except for Gazel, in which non-fatal asthma events were based on self-report from annual questionnaires and non-fatal COPD was not available. Asthma was defined as J45 or J46 (ICD-10) or 493 (ICD-9) in any diagnostic code.[9] COPD was defined as J41, J42, J43 and J44 (ICD-10) or 491, 492 and 496 (ICD-9).[10]

References

1. Nyberg ST, Fransson EI, Heikkila K, et al. Job strain as a risk factor for type 2 diabetes: a pooled analysis of 124,808 men and women. *Diabetes Care* 2014; **37**(8): 2268-75.
2. Kivimaki M, Hamer M, Batty GD, et al. Antidepressant medication use, weight gain, and risk of type 2 diabetes: a population-based study. *Diabetes Care* 2010; **33**(12): 2611-6.
3. Tabak AG, Jokela M, Akbaraly TN, Brunner EJ, Kivimaki M, Witte DR. Trajectories of glycaemia, insulin sensitivity, and insulin secretion before diagnosis of type 2 diabetes: an analysis from the Whitehall II study. *Lancet* 2009; **373**(9682): 2215-21.
4. Alberti KG, Zimmet PZ. Definition, diagnosis and classification of diabetes mellitus and its complications. Part 1: diagnosis and classification of diabetes mellitus provisional report of a WHO consultation. *Diabet Med* 1998; **15**(7): 539-53.
5. Tunstall-Pedoe H, Kuulasmaa K, Amouyel P, Arveiler D, Rajakangas AM, Pajak A. Myocardial infarction and coronary deaths in the World Health Organization MONICA Project. Registration procedures, event rates, and case-fatality rates in 38 populations from 21 countries in four continents. *Circulation* 1994; **90**(1): 583-612.

6. Kivimaki M, Nyberg ST, Batty GD, et al. Job strain as a risk factor for coronary heart disease: a collaborative meta-analysis of individual participant data. Lancet 2012; 380(9852): 1491-7.
7. Fransson EI, Nyberg ST, Heikkila K, et al. Job strain and the risk of stroke: an individual-participant data meta-analysis. *Stroke* 2015; **46**(2): 557-9.
8. Heikkila K, Nyberg ST, Theorell T, et al. Work stress and risk of cancer: meta-analysis of 5700 incident cancer events in 116,000 European men and women. *BMJ* 2013; **346**: f165.
9. Heikkila K, Madsen IE, Nyberg ST, et al. Job strain and the risk of severe asthma exacerbations: a meta-analysis of individual-participant data from 100 000 European men and women. *Allergy* 2014; **69**(6): 775-83.
10. Heikkila K, Madsen IE, Nyberg ST, et al. Job strain and COPD exacerbations: an individual-participant meta-analysis. *Eur Respir J* 2014; **44**(1): 247-51.

Supplement table 2. Incidence and mean age of diagnosis for individual noncommunicable diseases during follow-up in the pooled data.

Major chronic disease	Events	Incidence (per 10 000 person-years)	Mean age at diagnosis (years)
**Women**			
Type 2 diabetes	1683	26·4	57·9
CHD*	420	6·2	63·1
Stroke	516	7·8	58·2
Cancer	3331	51·5	55·9
Asthma or COPD	910	13·5	56·6
**Any of the above**	6137	96·5	56·3
**Men**			
Type 2 diabetes	2056	44·0	58·6
CHD*	1582	32·2	61·0
Stroke	750	15·3	61·4
Cancer	2922	61·1	61·5
Asthma or COPD	818	16·6	61·7
**Any of the above**	6797	149·6	59·8

*Myocardial infarction or cardiac death

The mean follow-up was 11·5 years (range between studies 6·3 – 18·6). In men, 8159 had at least one incident disease during 543 522 person-years at risk. The corresponding figure was 8100 for women during 785 350 person-years at risk. As expected, the incidence of each chronic disease was higher in men than women (Supplemental Table 2). However, except for CHD women were diagnosed on average at a younger age than men.

Appendix 3: Study-specific analyses

The studies in supplementary figures 1 and 2 are sorted by baseline year. There was some heterogeneity between study-specific estimates, I^2 varied between 7·1% and 94·0%. The highest variation was found in categories of normal weight, overweight and obesity class I. The heterogeneity was not related to the baseline year of the study. No heterogeneity was observed in obesity class II and III. Despite heterogeneity, the overall pattern of greater loss of disease-free years with higher obesity was evident.

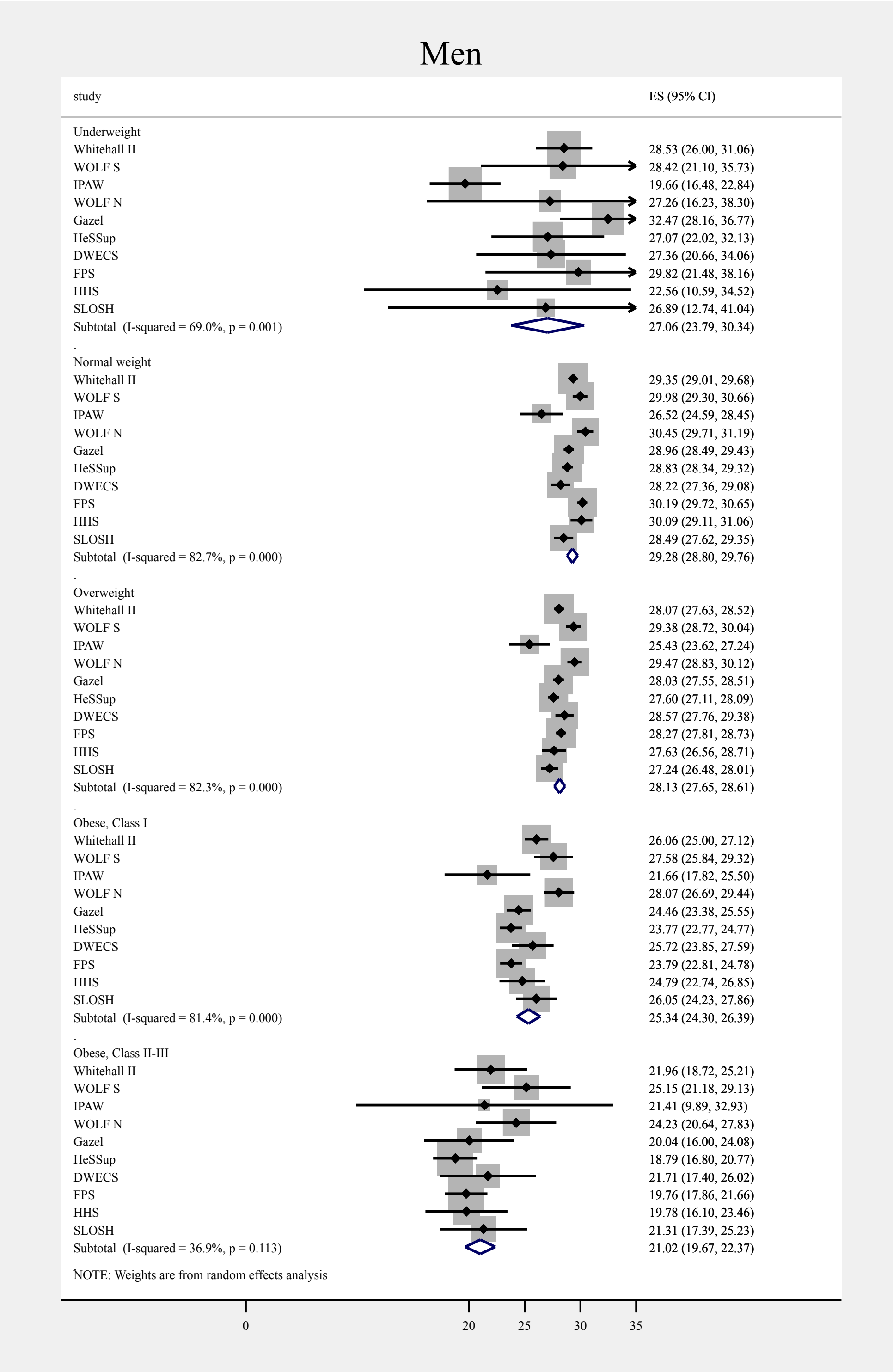

Supplemental figure 1 Study-specific estimates for men.

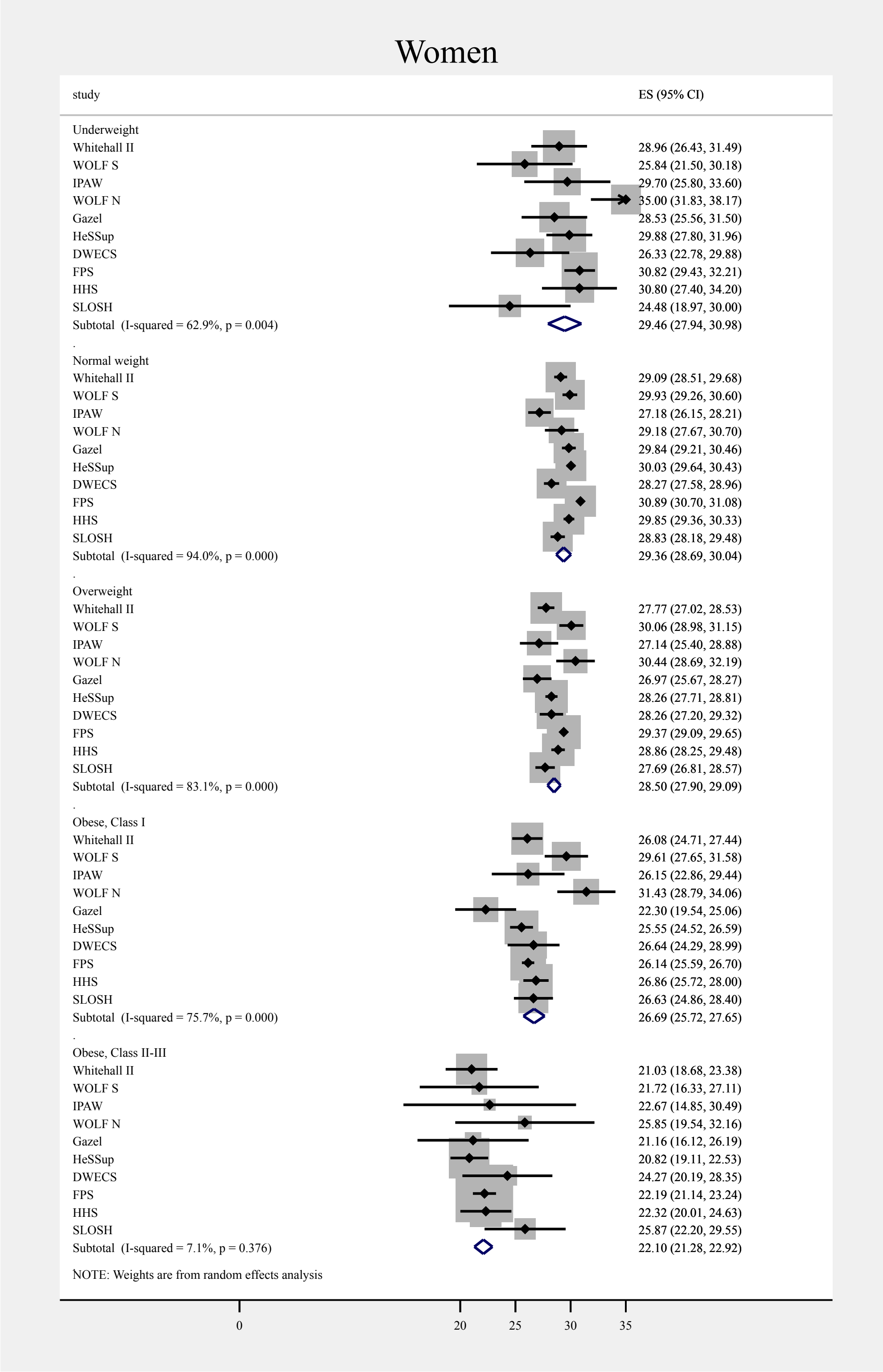

Supplemental figure 2. Study-specific estimates for women.

Appendix 4: Sensitivity and supplementary analyses

We first examined whether the use of pooled individual-level data across all studies was likely to be robust for a study of subgroups. To do so, we pooled the individual-level data and compared results from the pooled analysis with those obtained from the two-stage meta-analysis. Differences between estimates from the two analytic approaches were small (maximum 3·3 % - supplement table 3), suggesting that pooled analyses can be used in stratified analyses.

In the two-stage analyses, we first calculated the estimates for each study separately and in the second step we calculated the summary estimate over the results using random-effect meta-analysis. In the pooled analysis we pooled all available data and calculated the estimate, additionally adjusting the model for study. For comparison purposes, DWECS, IPAW, HHS and SLOSH are excluded from the results reported here, because only summary data were available for these studies.

Supplement table 3. Comparison of mean disease-free years after age 40 from the two-stage analysis and the pooled analysis

	BMI category				
	Normal	Overweight	Obesity, Class I	Obesity, Class II-III	Underweight
**Men**					
Pooled analysis	29.6	28.4	25.3	20.5	29.1
Two-stage analysis (difference, %)	29.6 (-0.1)	28.4 (0.1)	25.4 (-0.4)	21.1 (-2.5)	28.9 (0.7)
**Women**					
Pooled analysis	30.8	29.4	26.7	22.6	30.3
Two-stage analysis (difference, %)	29.9 (2.8)	28.8 (2.0)	26.8 (-0.1)	21.8 (3.3)	30.2 (0.4)

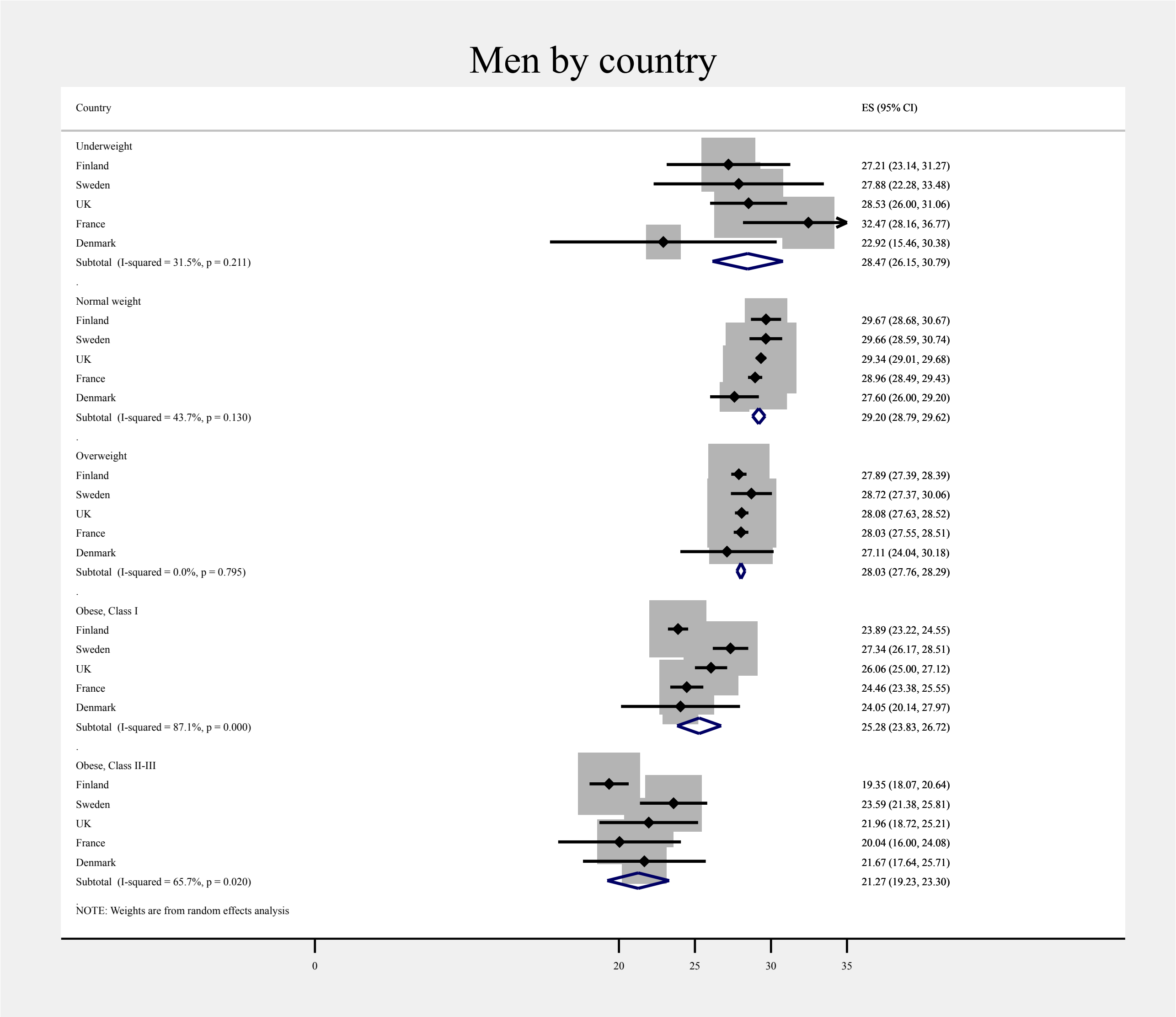

Supplementary figure 3. Country-specific estimates for the number of disease-free life-years after age 40 by BMI category for men.

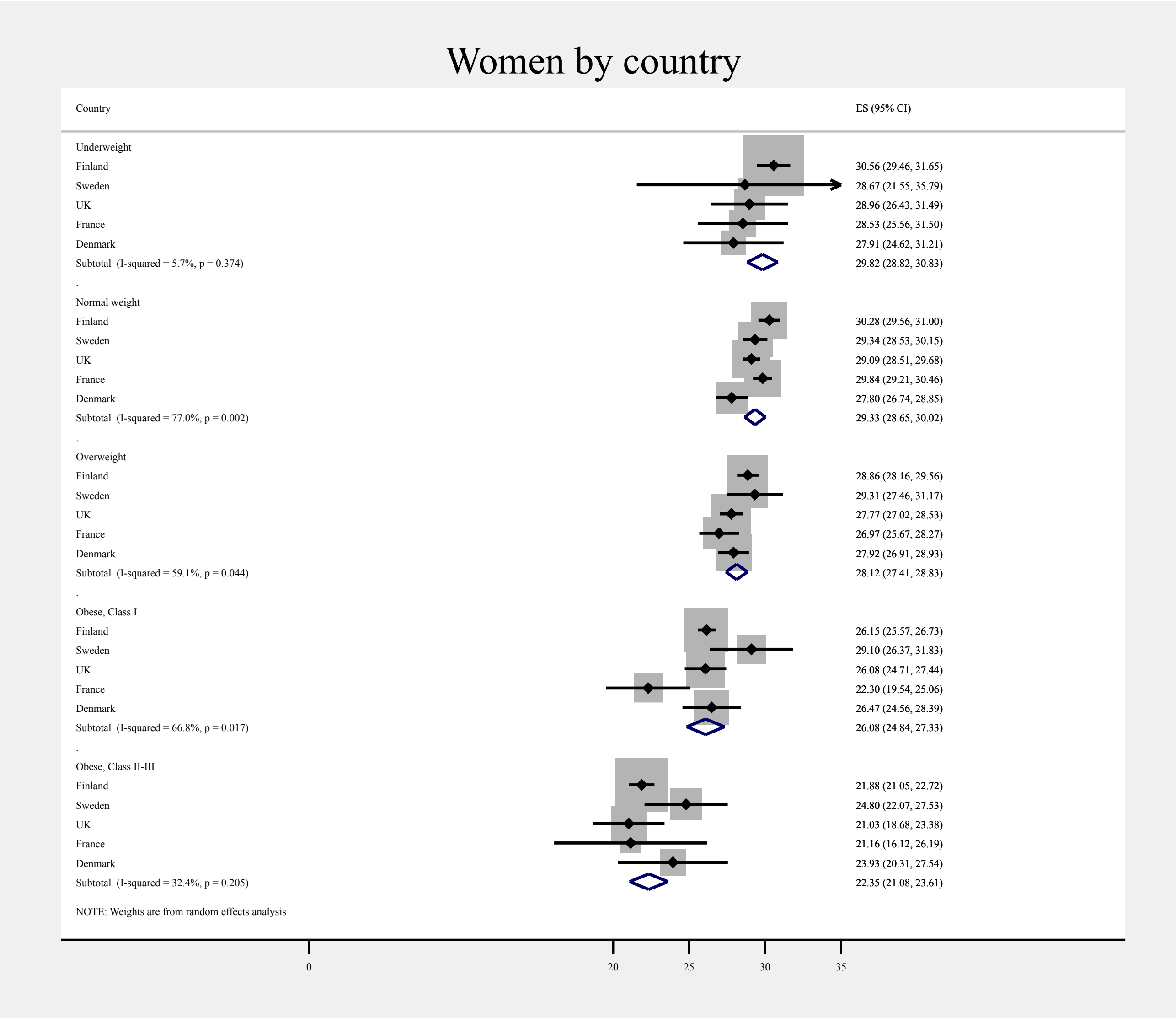

Supplementary figure 4. Country-specific estimates for the number of disease-free life-years after age 40 by BMI category for women.

Non-melanoma skin cancer does not often cause major lasting disability. We performed a sensitivity analysis in which non-melanoma skin cancer was not included in the definition of disease-free years. The findings did not change from the main analyses in which all cancers were included, corresponding to WHO definition of major non-communicable diseases (supplemental table 4).

Supplemental table 4. Number of disease-free life-years after age 40 by BMI category before and after excluding non-melanoma skin cancer has been excluded from the outcome

	**BMI category**				
	Normal	Overweight	Obesity, Class I	Obesity, Class II-III	Underweight
**Men**					
All cancers included	29.6	28.4	25.3	20.5	29.1
Non-melanoma skin cancer excluded	29.7	28.5	25.3	20.5	29.3
**Women**					
All cancers included	30.8	29.4	26.7	22.6	30.3
Non-melanoma skin cancer excluded	30.8	29.4	26.7	22.5	30.4

Supplementary table 5. Loss of disease-free years in underweight, overweight and obese participants compared to those with normal by smoking status, physical activity and socioeconomic position

	N	Disease-free life-years lost compared to normal weight				
		Underweight	Normal weight	Overweight	Obese, Class I	Obese, Class II-III
**Men**						
Total	42 622	0·3 (-1·5 to 2·1)	0·0 [reference]	1·2 (0·9 to 1·6)	4·0 (2·9 to 5·0)	8·6 (7·1 to 10·1)
Smoking status						
Never	13 757	0·2 (-2·3 to 2·7)	0·0 [reference]	1·0 (0·6 to 1·4)	3·7 (2·8 to 4·6)	7·1 (4·8 to 9·3)
Ex	13 127	-1·3 (-4·9 to 2·2)	0·0 [reference]	1·1 (0·6 to 1·5)	3·7 (3·0 to 4·5)	9·3 (7·6 to 11·0)
Current	7729	1·3 (-3·3 to 6·0)	0·0 [reference]	1·6 (1·0 to 2·2)	5·0 (3·8 to 6·2)	10·0 (7·9 to 12·2)
Physical activity						
Inactive	8554	1·8 (-2·3 to 5·9)	0·0 [reference]	0·7 (0·1 to 1·2)	3·6 (2·7 to 4·5)	8·8 (7·2 to 10·4)
Active	27 078	-0·5 (-2·6 to 1·7)	0·0 [reference]	1·3 (1·0 to 1·6)	4·1 (3·4 to 4·7)	8·0 (6·4 to 9·6)
Socioeconomic position						
High	10 921	1·4 (-2·3 to 5·1)	0·0 [reference]	1·3 (0·9 to 1·7)	3·6 (2·7 to 4·4)	8·7 (6·0 to 11·4)
Intermediate	14 387	0·7 (-2·2 to 3·6)	0·0 [reference]	1·1 (0·6 to 1·5)	4·7 (3·8 to 5·5)	10·2 (8·4 to 12·1)
Low	9852	-2·6 (-6·6 to 1·5)	0·0 [reference]	0·7 (0·1 to 1·3)	3·9 (2·8 to 4·9)	7·8 (6·0 to 9·5)
**Women**						
Total	67 778	0·0 (-1·6 to 1·7)	0·0 [reference]	1·2 (0·8 to 1·7)	2·9 (1·6 to 4·3)	7·8 (6·5 to 9·0)
Smoking status						
Never	26 251	-1·3 (-4·7 to 2·1)	0·0 [reference]	1·0 (0·6 to 1·4)	3·7 (2·9 to 4·4)	9·2 (7·5 to 10·8)
Ex	17 728	0·3 (-1·7 to 2·4)	0·0 [reference]	1·3 (0·8 to 1·8)	4·0 (3·2 to 4·8)	9·3 (7·9 to 10·7)
Current	10 123	1·0 (-1·2 to 3·2)	0·0 [reference]	1·7 (1·0 to 2·5)	4·3 (3·1 to 5·5)	8·9 (6·8 to 11·0)
Physical activity						
Inactive	11 269	1·2 (-0·9 to 3·3)	0·0 [reference]	1·4 (0·8 to 1·9)	3·6 (2·8 to 4·5)	8·4 (7·2 to 9·7)
Active	44 292	0·2 (-0·9 to 1·3)	0·0 [reference]	1·3 (1·0 to 1·6)	4·2 (3·6 to 4·8)	7·7 (6·5 to 8·9)
Socioeconomic position						
High	12 202	0·2 (-1·6 to 2·0)	0·0 [reference]	1·3 (0·7 to 1·8)	4·0 (2·9 to 5·2)	8·3 (6·1 to 10·6)
Intermediate	32 507	0·0 (-1·4 to 1·3)	0·0 [reference]	1·2 (0·8 to 1·6)	3·9 (3·2 to 4·6)	8·0 (6·8 to 9·1)
Low	11 006	1·7 (-0·4 to 3·9)	0·0 [reference]	1·9 (1·3 to 2·5)	4·2 (3·3 to 5·1)	8·0 (6·5 to 9·5)

Assessment of BMI using self-reported measurements of height and weight is vulnerable to subjectivity bias. We compared whether the association between BMI category and number of disease-free life-years differs by the method of BMI assessment. The association of obesity with disease-free life-years seems to be stronger in studies with measured rather than self-reported height and weight (supplementary figure 5).

Supplementary figure 5. Loss of disease-free years in overweight and obese participants compared to those with normal in studies with self-reported height and weight and those with measured height and weight.

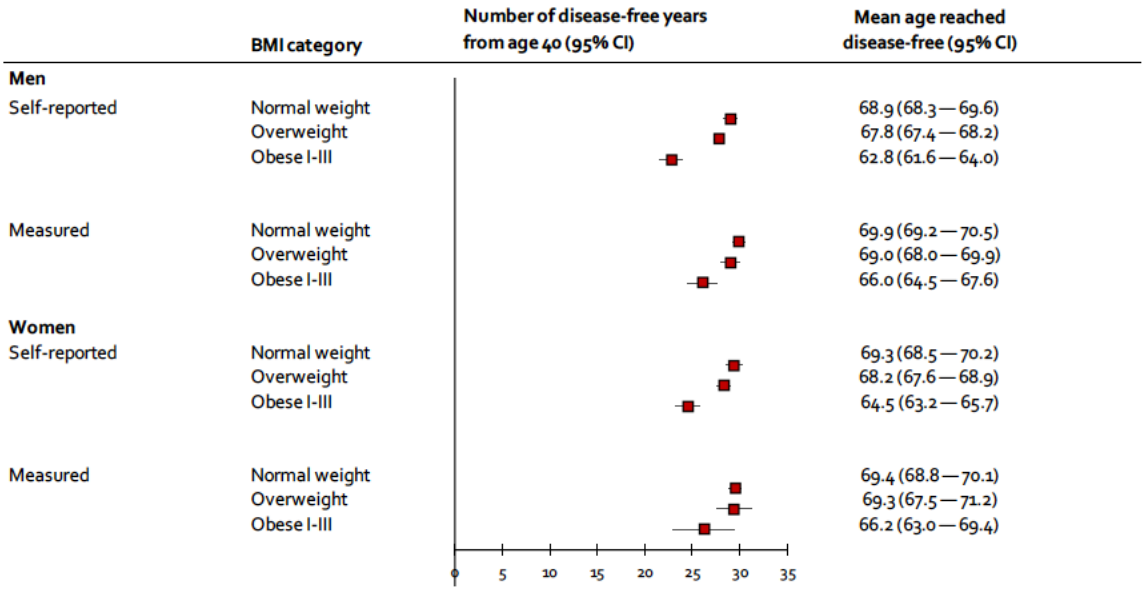

Supplement: Supplementary appendix [file mmc1.pdf]
